# Towards Precision Medicine for Osteoarthritis: Focus on the Synovial Fluid Proteome

**DOI:** 10.3390/ijms23179731

**Published:** 2022-08-27

**Authors:** Lorenzo Moretti, Davide Bizzoca, Alessandro Geronimo, Francesco Luca Moretti, Edoardo Monaco, Giuseppe Solarino, Biagio Moretti

**Affiliations:** 1 Orthopaedics Unit, Department of Basic Medical Science, Neuroscience and Sensory Organs, School of Medicine, University of Bari “Aldo Moro”, AOU Consorziale Policlinico, 70124 Bari, Italy; 2 PhD. Course in Public Health, Clinical Medicine and Oncology, University of Bari “Aldo Moro”, Piazza Giulio Cesare 11, 70124 Bari, Italy; 3 National Center for Chemicals, Cosmetic Products and Consumer Protection, National Institute of Health, Viale Regina Elena 299, 00161 Rome, Italy; 4 Orthopaedic and Trauma Surgery Unit, AOU Sant’Andrea, La Sapienza University of Rome, Via di Grottarossa 1035-1038, 00189 Rome, Italy

**Keywords:** proteomics, synovial fluid, osteoarthritis, biomarkers, precision medicine, medicine 4.0, joint, degenerative disease, musculoskeletal ageing

## Abstract

Osteoarthritis (OA) is a joint degenerative disease that most affects old age. The study of proteomics in synovial fluid (SF) has the task of providing additional elements to diagnose and predict the progress of OA. This review aims to identify the most significant biomarkers in the study of OA and to stimulate their routine use. Some of the major components of the ECM, such as proteoglycan aggrecan and decorin, were found considerably reduced in OA. Some biomarkers have proved useful for staging the temporality of OA: Periostin was found to be increased in early OA, while CRTA1 and MMPs were found to be increased in late OA. In its natural attempt at tissue regeneration, Collagen III was found to be increased in early OA while decreased in late OA. Some molecules studied in other areas, such as ZHX3 (oncological marker), LYVE1, and VEGF (lymph and angiogenesis markers), also have been found to be altered in OA. It also has been recorded that alteration of the hormonal pathway, using a dosage of PPAR-γ and RETN, can influence the evolution of OA. IL-1, one of the most investigated biomarkers in OA-SF, is not as reliable as a target of OA in recent studies. The study of biomarkers in SF appears to be, in combination with the clinical and radiological aspects, an additional weapon to address the diagnosis and staging of OA. Therefore, it can guide us more appropriately towards the indication of arthroplasty in patients with OA.

## 1. Introduction

Osteoarthritis (OA) is the most prevalent degenerative joint disease and a leading cause of disability and pain in elderly people.

OA has been defined by The Osteoarthritis Research Society International (OARSI) as “a disorder involving movable joints characterized by cell stress and extracellular matrix degradation initiated by micro-and macro-injury that activates maladaptive repair responses including pro-inflammatory pathways of innate immunity” [1].

OA represents a significant social health problem, affecting people worldwide, mainly among adults over 65 years [2].

Even if several genetic and environmental risk factors promote the development of OA, namely, age, body mass index (BMI), ethnicity, gender, physical activity, and muscle weakness, the exact pathogenesis of OA is still unclear [3]. The anatomopathological features of OA include cartilage degeneration, subchondral osteosclerosis, and intra-articular synovial inflammation [4].

Currently, the diagnosis of OA is based on a physical examination, while the progression of the joint degenerative changes can be assessed with imaging techniques only [3,4].

Osteoarthritis causes articular pain, joint stiffness, and loss of function, thus progressively affecting the patient’s working ability and social life [5]. Nonetheless, the onset of the symptoms occurs after the irreversible joint changes have developed [2]. Therefore, arthroplasty still is the most successful procedure for the treatment of OA, with a reported high patient satisfaction rate [6].

Thus, the search for synovial fluid (SF) biomarkers that could anticipate the diagnosis of OA is gaining increasing importance in orthopaedics [1]. In this context, the analysis of synovial fluid proteomics may play a central role in the future.

The proteome is the cell’s specific protein complement in a defined physiological context at a specific point in time [7]. Proteome analysis, or “proteomics”, is the large-scale protein-based systematic analysis of the proteome, or a defined sub-proteome, from a cell, tissue, or entire organism. It is a relatively new discipline that has been growing rapidly in the last two decades [8,9,10,11].

This systematic review aims to assess the current knowledge about the synovial fluid proteome in osteoarthritis. The proposed review sought to answer the following questions:

1. What is the correct sample processing for synovial fluid proteome analysis?
2. What is the potential of synovial fluid proteomics in clinical practice?

## 2. Material and Methods

The first step consisted of a scoping literature search performed by one reviewer, A.G., supervised by D.B., using the PubMed database to select an initial pool of potentially relevant papers, originally designed to investigate the role of the synovial fluid proteome in osteoarthritis. The search strategy included the following terms: ((“synovial proteome” [MeSH Terms] OR “synovial proteomics” [All Fields]) OR (“synovial proteome biomarkers” [MeSH Terms] OR “synovial proteins” [All Fields])) AND (“osteoarthritis” [MeSH Terms] OR (arthritis [All Fields])). The second step consisted of revising the literature review to identify papers dealing with the synovial proteome in osteoarthritis. Inclusion criteria were human studies in which the authors considered the role of the synovial proteome in osteoarthritis and those written in the English language. A total of 31 articles were finally included in the present review.

## 3. Synovial Fluid Physiopathology

Synovial fluid is a lubricant and functional plasma filtrate produced by the vascularized synovial membrane and surrounding cartilage and synovial tissue, composed of inflammatory proteins and cells. As suggested by Hollander et al. [12], routine synovial fluid analysis, in association with classic radiographic clinical evaluation, could help diagnose OA; the term “synovial analysis” was therefore introduced. The analysis of the synovial fluid appears to be an extremely useful diagnostic tool in the field of joint diseases since it provides valuable information on the staging and prognosis of OA. Recent scientific studies have shown the presence of different biomarkers present in the synovial fluid of patients with OA. Identification and the dosage of these biomarkers, which appears to be altered in patients suffering from OA, may, first of all, have a diagnostic role in the staging of the severity of osteoarthritis and therefore in predicting its evolution; secondly, it opens up possible scenarios of targeted therapy aimed at slowing down the evolution of this pathology, to procrastinate or even eliminate the need for arthroplasty.

Ali et al. showed in their study an overall higher protein expression in early-stage OA and a reduced response in late-stage OA [13].

## 4. Synovial Proteomic Biomarkers in OA

### 4.1. Cartilage Intermediate Layer Protein 1 (CILP)

Cartilage intermediate layer protein 1 (CILP) is a matrix protein that resides in the human articular cartilage and is involved in numerous diseases that affect cartilage. Its overexpression may lead to impaired chondrocyte growth and matrix repair [13,14,15,16]. Specifically, CILP has a relevant role in regulating the metabolism of the extracellular matrix (ECM). Moreover, it has been demonstrated that CILP can combine with transforming growth factor-β or insulin-like growth factor-1 to regulate the ECM synthesis of the intervertebral disc environment and so influence the balance of ECM metabolism, which leads to changes in the extracellular microenvironment, thus promoting the process of intervertebral disc degeneration [17,18].

### 4.2. Proteoglycan Aggrecan (ACAN or PGCA)

Proteoglycan aggrecan (ACAN or PGCA) is a major component of the ECM of cartilaginous tissues [13]. The importance of this protein is demonstrated by the various skeletal malformations that are created if its gene (which appears to be widely expressed during the growth period) is under-expressed, with consequently reduced deposition of aggrecan into the cartilage context. Moreover, aggrecan degradation is a hallmark of cartilage degeneration happening in OA. Studies on adult mice have shown that its hylomorphism is responsible for compromising the biomechanical properties of the joint surfaces, thus increasing the incidence of the osteoarthritis process [19,20]. In a recent open-labelled, noncontrolled clinical trial, recruiting patients suffering from knee osteoarthritis, the oral intake of resveratrol, at 500 mg/day for 90 days, induced a significant increase in the aggrecan serum level [19]. Concomitant pain and functional improvements, measured by the Visual Analogue Scale (VAS) and Knee Injury and Osteoarthritis Outcome Score (KOOS), were observed in these patients [19].

### 4.3. Decorin

Decorin, belonging to the SLRP family of the ECM, is a proteoglycan with the important function of binding the collage fibres together, as well as being one of the biomarkers most involved in the pathogenesis of OA. Both the aggrecan and decorin proteoglycan levels were found reduced in both early and advanced OA synovial fluid; this is explained by the increased catabolic activity and by an important remodelling action of the synovial tissue [13]. It has been demonstrated that there is a positive correlation between the decorin levels and the WOMAC Osteoarthritis Index, whose respective altered values and results were found to be risk factors for OA.

However, it was observed that the decorin levels in SF were not significantly altered in patients with OA [21]. In post-traumatic OA, decorin plays a protective role against cartilage degeneration, slowing down the loss of aggrecan and the fragmentation of the cartilage surface [22].

### 4.4. Periostin

Periostin, which also is associated with reparative processes involving cell adhesion and migration, was found to increase in both early- and late-stage OA in comparison to the controls, but there was a 3-fold difference in its levels when comparing early- and late-stage OA [13]. Recent evidence delineates an emerging role of periostin in OA since its expression after a knee injury is detrimental to the articular cartilage. It was seen how by inhibiting its expression on mice with post-traumatic OA, using a small interfering RNA (siRNA), significantly lower cartilage degeneration was obtained compared to the control cases. So, the prospect of using intraarticular delivery of the periostin–siRNA nano complex represents a promising clinical approach to mitigate the severity of joint degeneration in OA [23]. Other studies have pointed out how periostin deficiency protects against destabilization of the medial meniscus (DMM)-induced post-traumatic and age-related spontaneous OA [24].

### 4.5. Collagen Type III

Collagen is a major component of the ECM in cartilage. Particularly, collagen type III (CO3A1) was found in the Ali et al. study, increasing in early OA but decreasing in late-stage OA, compared to the controls. Collagen III is known to increase during regeneration and wound healing, suggesting an attempted regeneration process in early OA [13].

### 4.6. ZHX3

Due to the catabolic effect detected on the ECM proteins cited above, a compensatory increase in osteogenic differentiation also has been shown, represented by increased values of Zinc Fingers and Homeoboxes 3 (ZHX3) in OA patients, which is one of the most important biomarkers in oncology capable of predicting the outcome of cancer [13].

### 4.7. LYVE1

On the other hand, lymphatic vessel endothelial hyaluronic acid receptor 1 (LYVE1) was decreased in the OA patients in comparison to the controls, indicating a decreased lymphangiogenesis. Both proteins have a 2-fold change in differential expression in OA patients compared to the controls [1]. In favour of the above, this biomarker has also been tested to be used as a predictor of the clinical outcome of patients undergoing corrective osteotomies in patients with OA, thus helping to determine which patients will be best suited to treatment with osteotomy [25].

### 4.8. CRTAC1

Cartilage Acid Protein 1 (CRTAC1), a glycosylated ECM protein that can be found in the deep zone of articular cartilage, was found to be exclusively upregulated in late-stage OA, thus reflecting a severely damaged knee and a higher exposure of the synovial fluid to the deep zone of articular cartilage [13]. In a study done by Styrkarsdottir et al. [26], in search of a large panel of plasma proteins in patients with OA, it was noted that CRTAC1 was one of the main biomarkers most strongly associated with the diagnosis of OA and was found to be predictive of progression to total joint replacement. Patients with OA who were in the highest percentage of risk of joint replacement, based on the known risk factors (i.e., age, sex, and body mass index) and CRTAC1 plasma levels, were 16 times more likely to undergo knee arthroplasty within 5 years from the moment of plasma sampling than those in the lowest quintile [26]. It was also noted that CRTAC1 may be a sex-specific biomarker of OA progression in females [27].

### 4.9. PPAR-γ

An alteration in endocrine regulation by the inhibition of the peroxisome proliferator-activated receptors (PPAR-γ) has been established, which can play a role in the progression of OA. This transcription factor plays a central role in the regulation of lipid and glucose homeostasis; it is involved in the protein network through IGF and insulin-like growth factor binding proteins. IGF has previously been shown to play an important role in the regulation of glucose uptake by chondrocytes. So, this alteration triggers a metabolic disorder in chondrocyte proliferation [13]. Furthermore, the lack of PPAR in the synovial fluid could cause a worsening of OA, through the reduction of the chondroprotective function and, on the other hand, increasing the catabolic activity. This opens up possible therapeutic applications of PPAR-agonists, exploiting their ability to inhibit the production of inflammatory and catabolic factors involved in OA, and therefore reducing the development of cartilage alterations [28]. Interesting, to identify possible therapeutic targets to slow down the progress of OA, some studies have tried to find the candidate agents for the treatment of OA, such as oridonin (a diterpenoid isolated from *Rabdosia rubescens*) and mangiferin, which inhibit the IL-1β-induced inflammatory response in human osteoarthritis chondrocytes, acting as agonists of PPARs [29,30]

### 4.10. RETN

Again about the hormonal regulatory effect on OA, RETN is a hormone involved in glucose metabolism, having an indirect inhibitory action towards insulin by stimulating the absorption of glucose in the adipocytes; however, in OA synovial fluid, it has been related to increased catabolic activity in response to an inflammatory process. It has been seen how the inhibition of RETN in OA at the advanced stage leads to a reduction in the aforementioned catabolic activity [13]. RETN affects the OA risk and is associated with clinical features and severity in patients with OA [31].

### 4.11. Complement Component C8

Timur et al. [14] underlined that the complement system plays a key role in the pathogenesis of knee OA. In their study, the secretome of explanted OA meniscal tissue had the highest amount of complement component C8, thus suggesting that C8 might be put forward as a potential synovial fluid biomarker to evaluate the efficacy of therapeutic interventions in knee OA due to meniscal pathology [14]. Luo et al. [32] also measured the values of the complement component C8 in patients with osteoarthritis and type II diabetes mellitus (DM) and showed how its values were significantly increased in these patients. Many studies have shown the function of complement activation in the onset of metabolic diseases, including diabetes and obesity. As glycosylation of C8α N437 is involved in complement activation, it can therefore be deduced that the C8α complement activated in an altered manner in patients with DM may be involved in the cartilage degradation that occurs in OA. This explains the hypothesized correlation between DM and the increased severity of OA, therefore guiding us towards new therapeutic possibilities capable of inhibiting complement activation in OA patients with DM [32].

### 4.12. Clusterin

Clusterin (CLU), a protein that has been found overexpressed in the synovial fluid of patients with OA, was additionally demonstrated as being the most abundant in the explant secretomes of cartilage and meniscal tissues. This points towards the cartilage and meniscus as the tissues-of-origin of clusterin levels in OA synovial fluid [14]. Recent studies have shown that the clusterin levels are significantly higher in synovial fluid OA than in the control samples; a correspondence was also noted between its values in the blood and the synovial fluid. There was a markedly increased CLU level in synovial fluid and plasma in patients with advanced knee OA compared to patients with early-stage OA. A significant correlation was also observed between both the plasma and SF levels of CLU with the radiographic severity of knee OA. CLU was therefore found to be a novel biomarker for the diagnosis and staging of knee OA severity, with a sensitivity of 71.4% and a specificity of 73.3% [33,34,35].

### 4.13. IL-1

Yang et al. demonstrated in their study how many pro-inflammatory cytokines, namely, IL-1 and TNF-α, play a role in cartilage destruction and the progression of OA [15]. However, although it is well known that IL-1 is one of the two prototypical proinflammatory cytokines that participate in the pathogenesis of the degradation of the extracellular matrix and joint inflammation in OA, the quality of evidence for its involvement in disease is modest. While it is true that some in vivo studies have demonstrated elevated IL-1α and β values in OA synovial fluid and cartilage, on the other hand, several large double-blind, randomized controlled clinical studies targeting IL-1 have failed. Enthusiasm for IL-1 as a target in OA is rapidly dwindling [36,37].

### 4.14. TNF-Alpha

One of the pro-inflammatory cytokines that probably have a major role in inflammatory activation, cartilage damage, and the onset of pain in OA is tumour necrosis factor α (TNF-α). Recent studies have shown how the use of nonsteroidal anti-inflammatory drugs have a downregulation effect on TNF-alpha, thus ameliorating TNF-α-induced cellular senescence in human chondrocytes and therefore slowing down the progression of OA [38,39,40].

Zhang et al. demonstrated that the changes in epigenetic status regulate TNF-α expression in the cells, which are pivotal to the OA disease process [38].

### 4.15. Other Biomarkers

Giordano et al. [16] studied the levels of certain inflammatory biomarkers in the serum of patients with knee OA. Their study has shown that even a blood sample can provide us with information about the inflammatory state that OA generates and therefore also at a systemic level. They highlighted five cytokines with increased serum expression compared to the control cases; in particular, FGF-21 and 4E-BP1 were found to be related to pain specifically in patients with OA, while TWEAK, FGF-21, CSF-1, and IL-6 were identified as independent predictors for pain severity [4]. As far as cellularity is concerned, it was observed that, compared to healthy tissue, synovial tissue in OA patients contained a higher percentage of CD4 + T cells, memory B cells, mast cells, and dendritic cells [41]. Recent studies have highlighted new promising therapeutic targets for OA: for example, MMPs are involved in articular cartilage degradation in OA, but while often their SF values in patients with advanced OA were found to be significantly higher than those of patients with early-stage OA, in some cases they were not; they also were found to be related neither to the severity of OA nor, in some sporadic cases, to the presence of osteoarthritis itself [42,43,44,45].

VEGF was also found to have high values in OA-SF, also showing a positive correlation between its dosage and the clinical and radiographic severity of OA; this confirms the role of neoangiogenesis in the pathogenesis of OA [46,47,48,49,50,51,52,53,54].

Some controversial results have been found in the literature about emerging OA biomarkers. For example, COMP, which is a constituent of articular cartilage released from eroded cartilage, both in the SF and in the bloodstream, was found to be increased in the serum of patients with OA; however, these data have not been confirmed using the same dosage in SF, as most studies are unable to correlate the COMP values to the severity and prognosis of OA [48,50,51,52]. Similarly, CTX-II, which is a marker related to the degradation of type II collagen, was one of the most investigated molecules in urine samples, which led to a strong correlation with the severity of OA; however, these results were not confirmed later in studies where this biomarker was dosed in the SF [46,47,48,49,50,51,52,53,54,55].

## 5. Conclusions

Studies conducted on the research of biomarkers in OA synovial fluid have highlighted the important potential for diagnosing, predicting, and staging the severity of OA. Of the hundreds of molecules that have been investigated as part of proteomics research, we selected the ones that were found to be the most significant in terms of specificity and sensitivity for OA.

The finding of altered values of some of these molecules in different phases of OA has been demonstrated; for example, periostin appears to be increased more in early OA, while CRTAC1 and MMPs were found to be increased only in late OA.

Interestingly, some molecules were studied in other areas, such as the ZHX3 marker used in oncology for the prediction of the outcome of cancer; markers involved in the processes of lymphangiogenesis and neoangiogenesis, such as LYVE1 and VEGF, also have proven to be useful in predicting the evolution of OA.

Some of the molecules widely used in the past as targets for OA, such as IL-1, are turning out in the most recent studies to be not as reliable as OA markers, as previously was thought, and therefore the enthusiasm towards them is slowly decreasing.

On the other hand, the latest research has given rise to promising new biomarkers, such as FGF-21 and MMPs, the study of which can lead to greater chances of diagnosis and prognosis of OA.

In conclusion, we can confirm that the study of biomarkers in SF appears, in combination with the clinical and radiological aspects, an additional weapon to address the diagnosis and staging of OA. Therefore, it can guide us more appropriately towards the indication of arthroplasty in patients with OA. However, further studies are needed to standardize their use as a routine diagnostic approach to OA.

## Data Availability

Not applicable.

## Abbreviations

ACAN	Aggrecan
CLIP	Cartilage intermediate layer protein 1
CLU	Clusterin
CO3A1	Collagen type III
COMP	Cartilage Oligomeric Matrix Protein
CRTA1	Cartilage Acid Protein 1
CSF-1	Colony Stimulating Factor 1
CTX-II	C-telopeptide fragments of type II collagen
DM	Diabetes mellitus
DMM	Destabilization of the medial meniscus
ECM	Extracellular matrix
FGF-21	Fibroblast growth factor 21
IGF	Insulin-like growth factor
IL-1	Interleukin 1
KOOS	Knee Injury and Osteoarthritis Outcome Score
LYVE1	Lymphatic Vessel Endothelial Hyaluronic Acid Receptor 1
MMPs	Matrix Metalloproteinases
OA	Osteoarthritis
PGCA	Proteoglycan
PPAR- γ	Peroxisome proliferator-activated receptors γ
RETN	Resistin
SF	Synovial fluid
siRNA	Small interfering RNA
SLRP	Small Leucine-Rich Repeat Proteoglycan
TNF-alpha	Tumour Necrosis Factor—alpha
VAS	Visual Analogue Scale
VEGF	Vascular Endothelial Growth Factor
WOMAC	Western Ontario and McMaster Universities Arthritis Index
ZHX3	Zinc Fingers and Homeoboxes 3
